# Ischemic colitis in an infant with constipation treated with stimulant laxative

**DOI:** 10.1002/jgh3.12361

**Published:** 2020-05-25

**Authors:** Hirotaka Sakaguchi, Toshihiko Shirakawa, Tatsuki Mizuochi

**Affiliations:** ^1^ Department of Pediatrics and Child Health Kurume University School of Medicine Kurume Japan; ^2^ Department of Pediatrics Nagasaki University Hospital Nagasaki Japan

**Keywords:** children, constipation, ischemic colitis, stimulant laxative

## Abstract

Ischemic colitis (IC), the most common form of intestinal ischemia, ranges from superficial mucosal and submucosal injury to full‐thickness mural necrosis. As risk factors include cerebrovascular disease, hypertension, diabetes mellitus, prior abdominal surgery, irritable bowel syndrome, and constipation, IC typically occurs in elderly persons with multiple comorbidities rather than young children. A 1‐year‐old Japanese girl receiving a stimulant laxative for constipation since age 7 months was hospitalized for fever, vomiting, and hypovolemic shock. Her abdomen was swollen, and abdominal computed tomography showed colonic distension with abundant stool. Colonic decompression and intensive care brought about rapid improvement until persistent bloody diarrhea that commenced on day 17 of illness required transfer to another hospital, where colonoscopy on day 42 showed mucosal sloughing forming pseudomembranes, as well as focal stenosis. Contrast enema on day 45 confirmed stenosis with a “thumbprint” contour at the splenic flexure. Diagnosed with IC, she received parenteral nutrition and an elemental diet. Bloody diarrhea resolved by day 75. Colonoscopy and contrast enema on day 110 showed normal mucosa and resolution of stenosis. We believe that IC arose from constipation and stimulant laxative treatment and consider this to be the first report of infantile IC complicating constipation.

## Graphical abstract

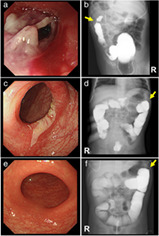

The manuscript describes a Japanese infant who was diagnosed with ischemic colitis caused by constipation and stimulant laxative treatment. To our knowledge, this is the first report of infantile ischemic colitis complicating constipation.

## Introduction

Considering all ages, ischemic colitis (IC) is the most frequent type of intestinal ischemia, which can range from superficial mucosal and submucosal injury to full‐thickness mural necrosis. Usually transient and self‐limited, IC can progress to gangrenous necrosis, causing perforation and peritonitis.^1^ Risk factors for IC typically accompany advancing age and include cerebrovascular disease, hypertension, diabetes mellitus, prior abdominal surgery, irritable bowel syndrome, and constipation,^2^ so IC is rare in children. Here, we report an infant with IC that complicated constipation treated with laxatives.

## Case report

**Figure 1:**
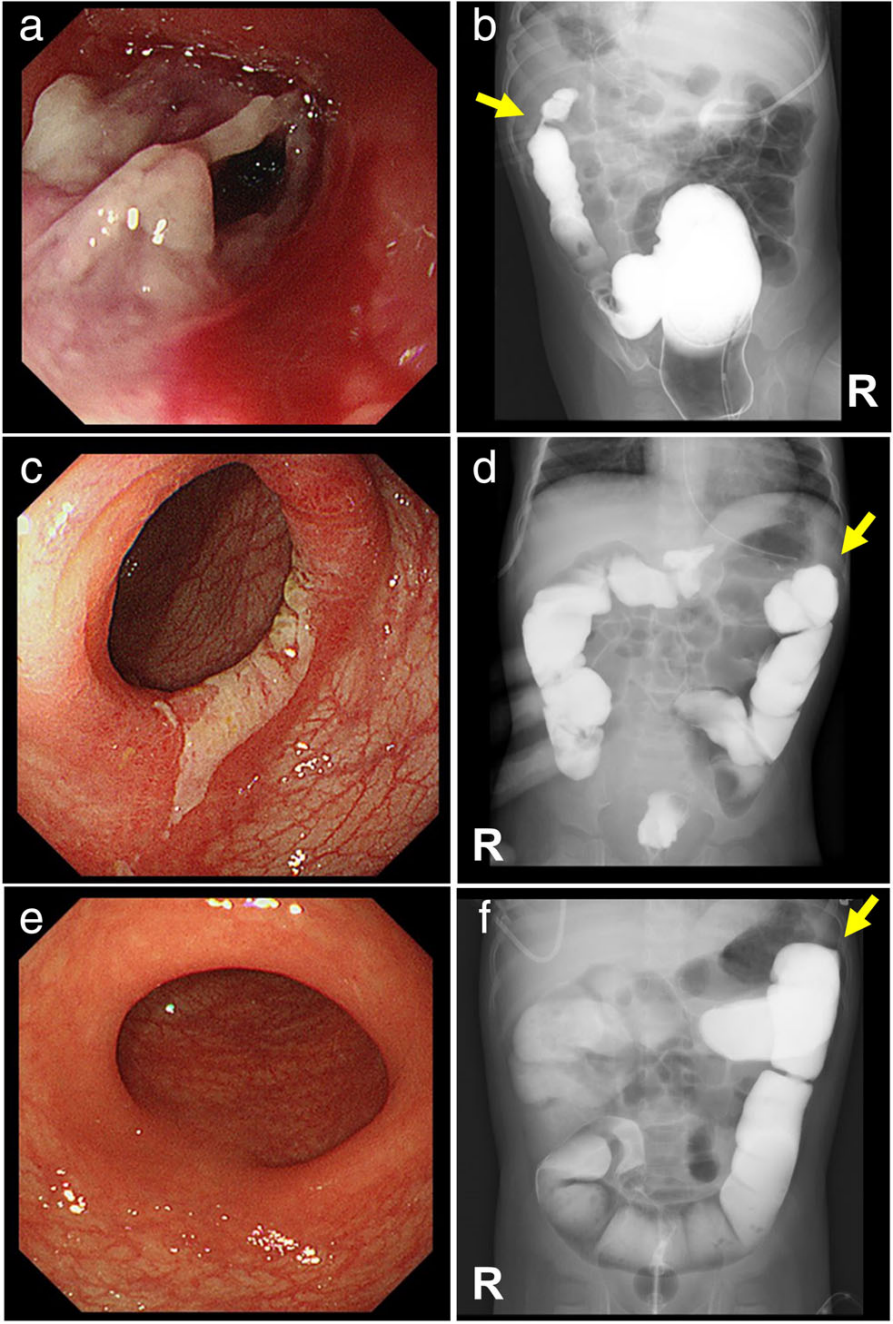
Colonoscopy and contrast enema findings. Colonoscopy on day 42 of illness (a) showed development of pseudomembranes related to mucosal sloughing. Contrast enema on day 45 (b) visualized focal stenosis from a stenotic lesion with a “thumbprint” contour at the splenic flexure. Colonoscopy (c) and contrast enema (d) on day 61 showed improvement of the stenotic lesion. Colonoscopy (e) and contrast enema (f) on day 110, respectively, showed normal colonic mucosa and full resolution of stenosis. R, right; arrows, splenic flexure.

A 1‐year‐old Japanese girl receiving a stimulant laxative for constipation since age 7 months was transported to Nagasaki University Hospital with fever, vomiting, and hypovolemic shock. Her abdomen was distended, and abdominal computed tomography showed colonic distension with abundant stool (Fig. S1a, Supporting information). Radiographs showed water‐soluble enema contrast filling the entire colon, excluding stenoses (Fig. S1b). Colonic decompression was followed by intensive care, with rapid improvement until persistent bloody diarrhea commenced on day 17 of illness, necessitating transfer to Kurume University Hospital. Colonoscopy on day 42 showed mucosal sloughing forming pseudomembranes, as well as focal stenosis (Fig. 1a). Contrast enema on day 45 showed stenosis with a “thumbprint” appearance at the splenic flexure (Fig. 1b). Examination of a rectal biopsy specimen excluded Hirschsprung's disease. Diagnosed with IC complicating constipation, the patient was treated with parenteral nutrition and an elemental diet. Colonoscopy and contrast enema on day 61 showed improvement of the stenotic lesion (Fig. 1c,d). Enteral nutrition and baby food were well tolerated, without symptoms of constipation in the absence of laxatives. Bloody diarrhea resolved by day 75. Colonoscopy and contrast enema on day 110 showed normal colonic mucosa and no stenoses (Fig. 1e,f).

## Discussion

As risk factors include cerebrovascular disease, hypertension, diabetes mellitus, prior abdominal surgery, irritable bowel syndrome, and constipation, IC usually occurs in elderly individuals with multiple comorbidities; pediatric reports are few.^2^ Constipation increases intraluminal pressure, reducing blood flow to colonic mucosa and favoring regional mural ischemia.^1^ IC associated with stimulant laxatives is believed to result from stimulated colonic motility increasing intraluminal pressure, compressing the splanchnic circulation to decrease colonic perfusion.^3^ We therefore believe that the patient's IC arose from constipation and stimulant laxative treatment. To our knowledge, this is the first report of infantile IC complicating constipation.

## Supporting information


**Figure S1** Abdominal computed tomography and contrast enema findings on day 1 of illness. (a) Abdominal computed tomography showed colonic distension with abundant stool. (b) Contrast enema filled the entire colon, showing no stenoses. R, right; arrow, splenic flexure.
