# Supplementary figures and images for: Ischemic colitis in an infant with constipation treated with stimulant laxative
Source: JGH Open. 2020 May 25;4(5):1012–3. doi: 10.1002/jgh3.12361 (PMC7578304; doi:10.1002/jgh3.12361)

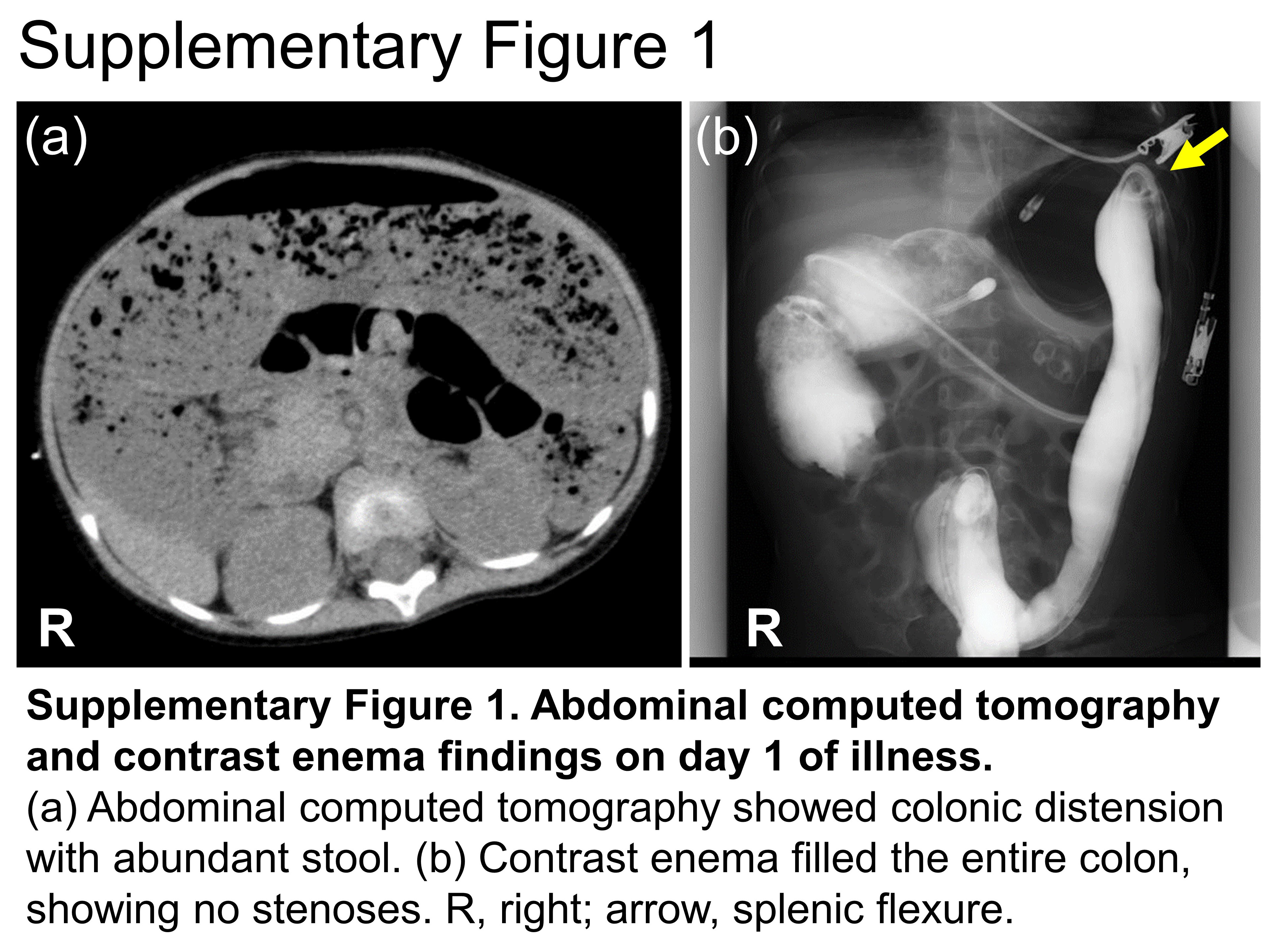

Supplement: Supplementary file 1 — Figure S1 Abdominal computed tomography and contrast enema findings on day 1 of illness. (a) Abdominal computed tomography showed colonic distension with abundant stool. (b) Contrast enema filled the entire colon, showing no stenoses. R, right; arrow, splenic flexure. [file JGH3-4-1012-s001.jpg]
